# Optimal Segment Selection on Gadoxetic Acid-Enhanced MRI to Improve Diagnostic Accuracy in the Histological Grading of Liver Inflammation and Fibrosis in Patients with Chronic Hepatitis B

**DOI:** 10.3390/jcm14228025

**Published:** 2025-11-12

**Authors:** Korcan Aysun Gonen, Mehmet Fatih Inecikli, Rafet Mete, Meltem Oznur

**Affiliations:** 1 Department of Radiology, Tekirdag Namık Kemal University School of Medicine, 59100 Tekirdag, Turkey; 2 Department of Radiology, Bursa Uludag University School of Medicine, 16059 Bursa, Turkey; drmfinecikli@uludag.edu.tr; 3 Department of Gastroenterology, Tekirdag Namık Kemal University School of Medicine, 59100 Tekirdag, Turkey; rmete@nku.edu.tr; 4 Department of Pathology, Tekirdag Namık Kemal University School of Medicine, 59100 Tekirdag, Turkey; moznur@nku.edu.tr

**Keywords:** Gadoxetic acid, magnetic resonance imaging, signal intensity, liver fibrosis, chronic hepatitis B

## Abstract

**Background/Objectives:** To investigate the role of hepatobiliary phase (HBP) signal intensity (SI) on Gadoxetic acid (GA)-enhanced liver magnetic resonance imaging (MRI) in improving the diagnostic accuracy of the histological grade of fibrosis in patients with chronic hepatitis B (CHB). **Methods:** This retrospective study enrolled patients with CHB who underwent biopsies from the highest and lowest intensity areas identified on HBP images obtained from GA-enhanced MRI. The patients were divided into two groups based on segmental SIs: Group 1 (maximum SI) and Group 2 (minimum SI). An ultrasound-guided tru-cut biopsy was performed in these two segments. Forty patients undergoing histopathological examination were included in the study. Group comparisons were examined using Chi-square and independent-sample *t*-tests, and receiver operating characteristic curve analysis (ROC) was performed to determine the cutoff values of the SI for modified histologic activity index (mHAI) and fibrosis grading. **Results:** There were no histopathological differences between the groups (*p* > 0.05), but significant inflammation and fibrosis were observed in hepatic segments with an SI value of <617 (*p* < 0.001). The ROC results showed that the predictive cutoff value of SI for mHAI and fibrosis grading were 606 (AUC: 0.83, 95% CI 0.737–0.921, *p* < 0.001) and 599 (AUC: 0.85, 95% CI 0.766–0.935, *p* < 0.001), respectively. **Conclusions:** In patients with CHB, performing a biopsy from the liver segment with the lowest SI on GA-enhanced MRI increases the diagnostic accuracy for assessing the histological severity of hepatic inflammation and fibrosis.

## 1. Introduction

In patients with chronic hepatitis B (CHB), progression of liver fibrosis may result in cirrhosis, portal hypertension, liver failure, and hepatocellular carcinoma (HCC). Early antiviral therapy can prevent these complications, increase survival rates, and reduce the need for liver transplantation. Antiviral therapy is recommended for patients with significant fibrosis (fibrosis stage 2) to halt the progression of fibrosis [1,2]. Therefore, the assessment of liver fibrosis in CHB is extremely important, and accurate and timely staging of fibrosis is of vital significance [3,4,5].

Noninvasive biochemical markers and imaging techniques are commonly used to assess the degree of liver fibrosis. However, routine biochemical/hematological tests and certain serological test algorithms are insufficient to detect fibrosis in approximately half of the patients, particularly those with moderate to advanced stages [1,6,7]. Ultrasound (US) elastography and magnetic resonance elastography (MRE) are the most commonly used imaging techniques for the noninvasive assessment of liver fibrosis. Both methods can only indirectly reflect liver stiffness and fibrosis. US elastography offers high reproducibility and reliability; however, its field of view is limited, and obtaining consistent measurements requires the operator to be adequately trained and experienced. MRE demonstrates the best performance in the evaluation of liver fibrosis and shows good correlation with fibrosis severity. Nevertheless, this method also requires passive acoustic drivers and specialized software to generate mechanical waves. Therefore, its accessibility is limited, and its use in routine clinical practice is challenging [1,8]. It has been demonstrated that there is a significant negative correlation between the apparent diffusion coefficient (ADC) obtained from diffusion-weighted imaging (DWI), another prominent method for fibrosis assessment, and the stage of fibrosis [9].

Gadoxetic acid (GA) [Eovist or Primovist, Bayer HealthCare, Berlin, Germany] is a hepatocyte-specific extracellular magnetic resonance imaging (MRI) contrast agent. It is widely used for the evaluation of focal liver lesions and the detection of liver fibrosis [4,10,11]. The contrast enhancement index (CEI) calculated from GA-enhanced MRI is considered a more effective biomarker for staging liver fibrosis compared to certain hematological parameters and ADC values [3]. Acquisition of GA-enhanced MRI images does not require specialized equipment or advanced analysis software, nor does performing region of interest (ROI) measurements for fibrosis assessment require extensive operator experience. In this respect, it is a method that can be easily used in daily routine at any MRI center.

Despite the increasing use of imaging techniques, liver biopsy is still considered the gold standard for assessing liver inflammation and fibrosis in CHB [3,12]. However, the most frequently cited disadvantage of this method is its limitation due to sampling error, as it is performed randomly [1,6,8]. Histopathological evaluation, which is generally based on a small tissue sample of 10–15 mg, may not adequately represent the entire liver, which weighs approximately 1500 g [12,13,14].

Among available MRI contrast agents, GA shows the highest hepatocyte uptake, thereby offering the greatest potential for assessing hepatocyte function [4,5]. However, studies on GA-enhanced MRI have shown that GA does not distribute evenly across all regions of the liver [3,4]. Furthermore, in liver diseases with non-focal and heterogeneous distribution, such as chronic viral hepatitis, the reliability of biopsy is limited by sampling variability [15,16]. Determination of regions based on signal intensity (SI) measurements on GA-enhanced MRI can guide biopsies towards areas that better represent the liver, thereby helping to avoid random biopsies. This approach can improve the accuracy of histopathological staging and enhance diagnostic reliability in patients with diffuse liver disease. Therefore, the aim of this study is to evaluate the use of regional SI measurements on GA-enhanced MRI as a guide for biopsy site selection to most accurately stage liver fibrosis in patients with CHB.

## 2. Materials and Methods

This single-center retrospective study included patients with CHB who underwent biopsies from the highest and lowest intensity areas identified on hepatobiliary phase (HBP) images obtained from GA-enhanced MRI. Over a 10-year period, a total of 4368 consecutive liver biopsies were performed in our interventional radiology (IR) unit. Of these, 3083 were parenchymal biopsies. Among these patients, those who met the following criteria were investigated: (a) had CHB, indicated by HBsAg positivity persisting for over six months; (b) had liver MRI enhanced with GA; (c) had no previous history of HCC and other malignancies; (d) had no previous history of hepatitis treatment; (e) had no previous history of other parenchymal diseases such as viral hepatitis C, primary biliary cirrhosis, autoimmune hepatitis, or Wilson disease; (f) had SI measurements of each segment on HBP images. Following this detailed review, 86 patients were included for further evaluation. The final exclusion criteria for the 86 patients were as follows: incomplete MRI examination (*n* = 17), inability to access MRI scans (*n* = 11), refusal to undergo double-segment biopsy (*n* = 17), and failure to perform pathological examination in at least one of the two segments (*n* = 1). Ultimately, 80 biopsy procedures performed on 40 patients were included in the study. This study was approved by the Institutional Review Board (ID number: 2019.165.09.25) and adhered to the Declaration of Helsinki. Written informed consent was obtained from all patients.

### 2.1. MRI

MRI images were obtained using a GE Optima 360 1.5 T scanner (General Electric Medical Systems, Milwaukee, WI, USA). The patients were fasted for 8–10 h before the MR examination. Abdominal images were obtained in the axial plane using an 8-channel torso coil. The scanning range extended from just above the upper margin of the liver to below the lower edges of both kidneys. The routine liver MR protocol included non-enhanced fast spin-echo T1-weighted (T1WI), fast spin-echo T2-weighted (T2WI), DWI, and enhanced T1WI. The contrast agent was used at 0.025 mmol per kilogram of body weight GA. This was followed by a 15–20 mL flush with physiological saline. The contrast-enhanced protocol consisted of a three-dimensional volumetric interpolated breath-hold T1-weighted LAVA sequence in the axial plane (repetition time, 4.01 msec; echo time, 1.9 msec; flip angle, 120; section thickness, 4.6 mm; intersection gap, zero; matrix 320 × 192; field of view, 400 mm). For each patient, routine triphasic MRI (arterial, portal, equilibrium phases) and HBP images were obtained. Arterial phase images were obtained approximately 20 s after beginning the contrast bolus injection at 2 mL/s, followed by portal phase, equilibrium phase, and HBP images (60 s, 3 min, and 20 min after injection, respectively).

### 2.2. Image Analysis and Biopsy Procedure

**Figure 1:**
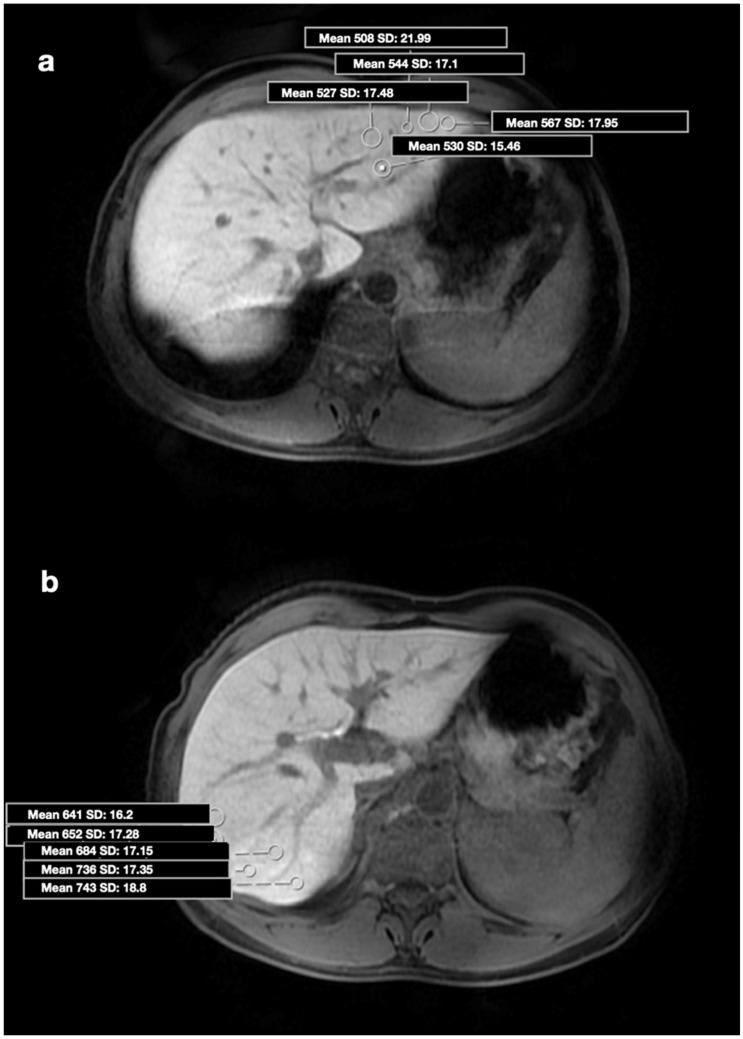
(**a**) Axial HBP MR image shows placement of ROIs in the 2nd segment (Histopathology—mHAI: 5/18; fibrosis: 2/6). HBP: Hepatobiliary phase, ROI: region of interest, mHAI: modified histologic activity index. (**b**) Axial HBP MR image shows placement of ROIs in the 7th segment (Histopathology—mHAI: 3/18; fibrosis: 1/6). HBP: Hepatobiliary phase, ROI: region of interest, mHAI: modified histologic activity index.

**Figure 2:**
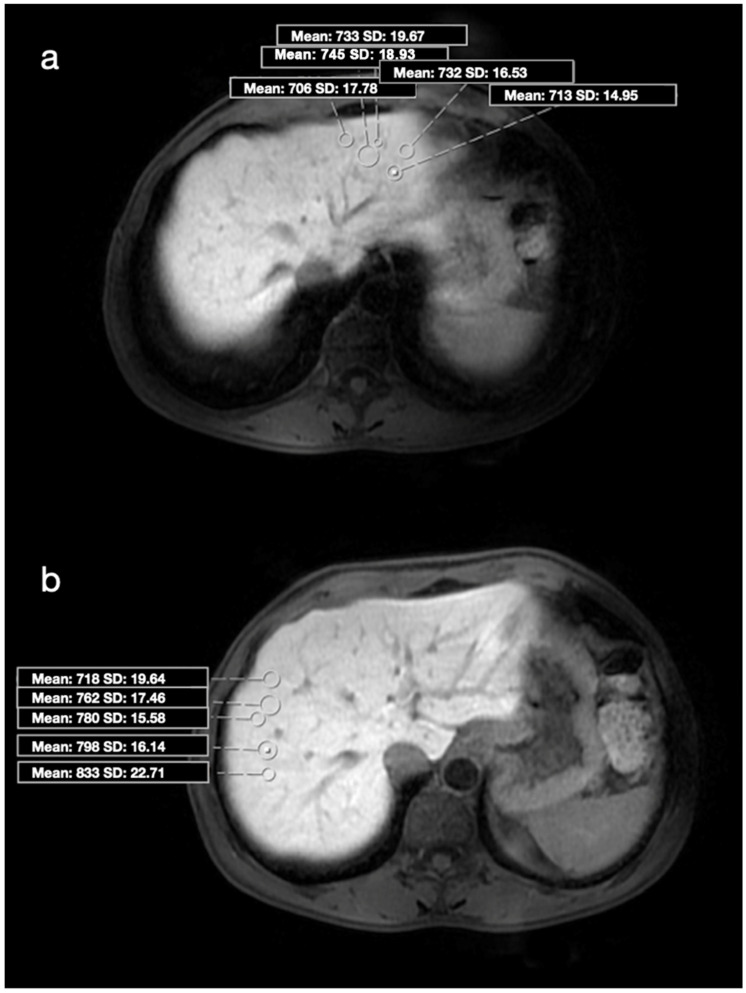
(**a**) Axial HBP MR image shows placement of ROIs in the 2nd segment (Histopathology—HAI, 2/18; fibrosis, 0/6). HBP: Hepatobiliary phase, ROI: region of interest, mHAI: modified histologic activity index. (**b**) Axial HBP MR image shows placement of ROIs in the 8th segment (Histopathology—mHAI, 2/18; fibrosis, 0/6). HBP: Hepatobiliary phase, ROI: region of interest, mHAI: modified histologic activity index.

**Figure 3:**
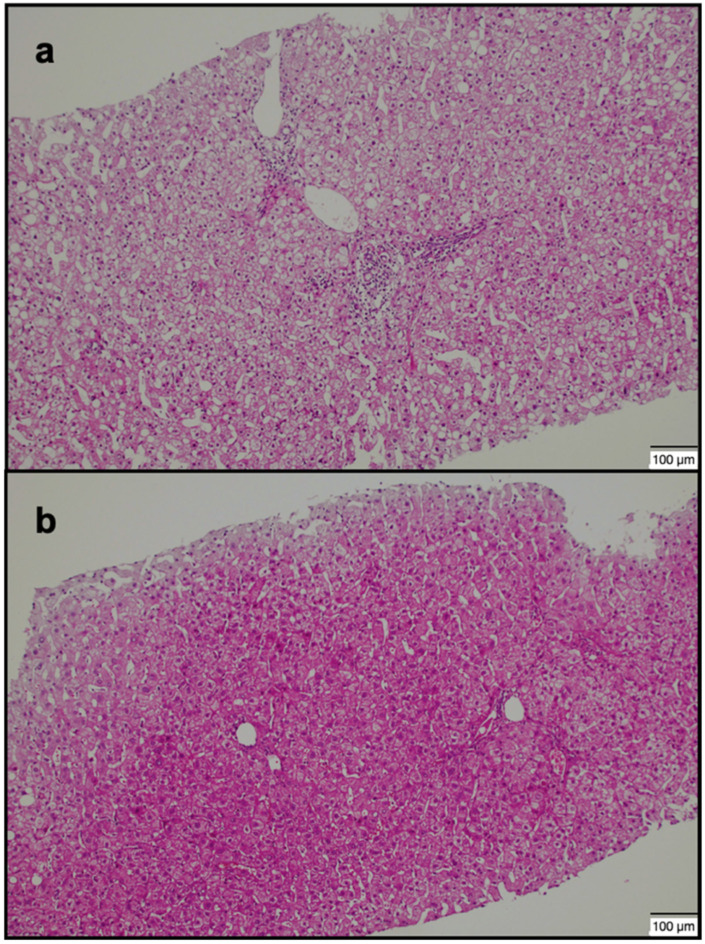
A 63-year-old female patient: (**a**) Moderate inflammation and mild fibrosis in the 2nd segment (lowest SI) (H&E. × 100). (Grade 2: mHAI, 5/18; fibrosis, 2/6). (**b**) Minimal inflammation in the 7th segment (highest SI) (H&E. × 100). (Grade 1: mHAI:3/18; fibrosis:1/6). *The pathology of the patient in* Figure 1. SI: signal intensity; mHAI: modified histologic activity index.

**Figure 4:**
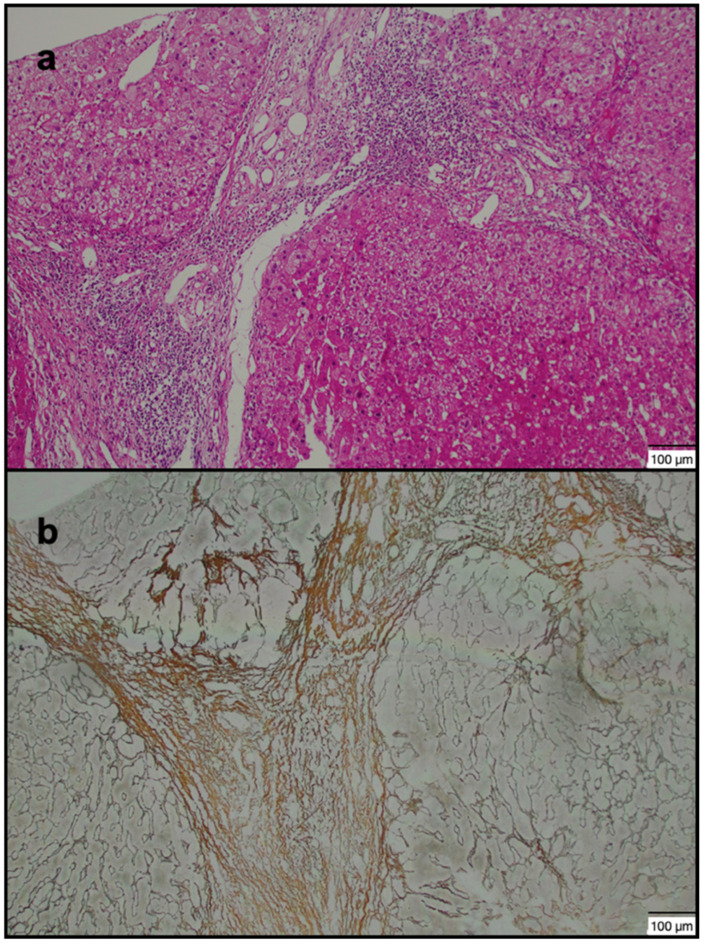
A 40-year-old male patient: (**a**) Severe inflammation and marked fibrosis in segments 2 (lowest SI) and 5 (highest SI) of the liver (H&E. × 100). (**b**) Marked fibrosis with reticulin staining in the same patient (Reticulin stain. × 100). (Grade 4: mHAI: 13/18; fibrosis: 4/6). SI: signal intensity; mHAI: modified histologic activity index.

MR images were transferred to the Vitrea 2 workstation (Vital Images, Canon, Minnetonka, MN, USA), and evaluations were made on axial HBP images. Imaging analysis was performed by a radiologist with 15 years of experience in the abdominal region, who was blinded to the patients’ clinical data. All images of the patients with a history of CHB were analyzed in detail due to the risk of additional pathologies. ROI was drawn manually as round shapes on the liver in HBP images (mean: 20 mm^2^), and SI measurements were made from almost every point (a minimum of 4 to 5 different foci) of each segment (Figure 1a,b and Figure 2a,b). ROIs were placed carefully to avoid positioning over a possible cyst, biliary ducts, portal, or hepatic veins. Subsequently, the mean SI value for each segment was calculated as the average of all ROIs within that segment. Based on the calculated mean SI values, two segments with the highest (group 1 = maximum SI) and lowest (group 2 = minimum SI) SI values were identified in each patient’s liver. Additionally, the median SI value across all segments was calculated, and two further groups were defined according to whether the SI values were above or below 617 (SI values > 617 and <617). For 41 patients who accepted the biopsy procedure, the procedure plan was made in accordance with our institutional protocol. Anticoagulant medications were discontinued one week before the procedure, and coagulation parameters were checked one day prior to the intervention. All biopsy procedures were performed on an outpatient basis. Patients were kept fasting for 10–12 h before the procedure and were monitored throughout the procedure. To determine the biopsy sites, GA-enhanced MRI images were opened on the workstation in the biopsy room. On the HBP images, the segments previously recorded as having maximum and minimum SI were carefully reviewed. With the patient in the supine position, a rapid general evaluation of the liver was performed using US, and the biopsy regions were determined under MRI guidance. No patient received general anesthesia or sedation during the procedure; 2% prilocaine hydrochloride was administered to all patients as local anesthesia. The US-guided percutaneous biopsies were performed by an experienced radiologist who was blinded to patient data (with 12 years of experience in liver biopsy) from 2 different segments (with the maximum and minimum SI values) of the liver. Full-automatic Bard needles (CR Bard, Inc., Covington, GA, USA) with a 2 cm shot distance were used for the procedure. The obtained tissue samples were fixed in formalin and embedded in paraffin. Histopathological evaluation was performed by a pathologist who was experienced in hepatic histopathology for more than 10 years. She was blinded to the MR image analysis and patient data. Pathological examination was performed using a modified histologic activity index (mHAI) and Ishak–Knodell Scoring System, and inflammation and fibrosis scoring were assessed [17]. Based on that, the clinically significant activity limit was accepted as ≥5 for grades 1–18, and ≥2 for fibrosis staging of 1–6 (Figure 3a,b and Figure 4a,b). After the procedure, patients were observed for 3 h with a 5 kg sandbag placed over the biopsy site. During this period, the monitored patients underwent close monitoring of blood pressure and pulse. After three hours, patients with normal hemodynamic findings and stable clinical conditions were allowed to have oral intake and discharged. Before discharge, all patients underwent a complete abdominal US examination to rule out possible bleeding complications; in particular, the intrahepatic area, perihepatic region, and all abdominal quadrants were evaluated for the presence of free fluid. No minor or major complications developed during or after the procedure. Patients were instructed to lie on their abdominal region for 12 h after discharge except for daily necessities, and to avoid taking any anticoagulant medications for one week. They were advised to contact our clinic in case of any signs of complications, and were scheduled for a follow-up visit one week later. At the follow-up, patients were evaluated for possible complications such as bleeding or infection, and a repeat abdominal US was performed.

### 2.3. Statistical Analysis

Statistical analysis was performed with SPSS for Windows 22.0 (Statistical Product and Service Solutions, Inc., Chicago, IL, USA) package program. In the descriptive analysis, the measurement variables are presented as a mean ± standard error. The differences between groups were assessed using Chi-square and independent sample *t*-tests. The diagnostic value of the SI measurement for the evaluation of the mhai (≥5) and fibrosis score (≥2) was assessed using receiver operating characteristic (ROC) curve analysis. The optimal cutoff points were determined by the Youden index as follows: Sensitivity—(1—Specificity). The area under the curve (AUC) was calculated from the ROC curves. *p* < 0.05 was considered statistically significant.

## 3. Results

**Figure 5:**
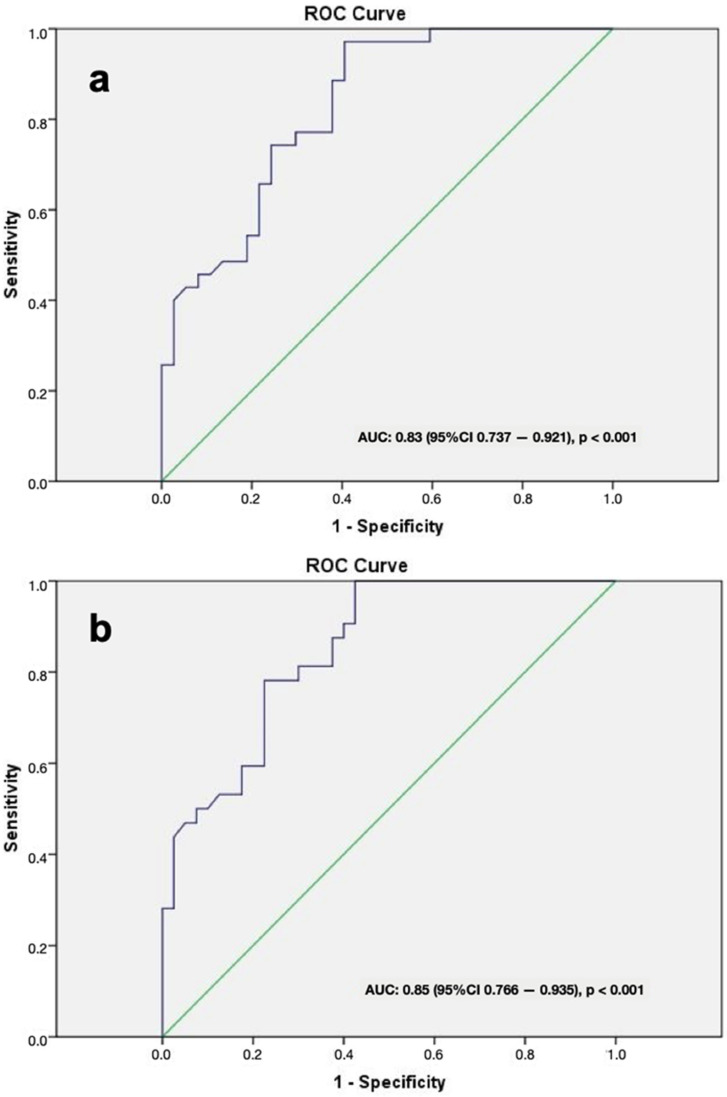
(**a**) ROC curves for detecting mHAI in patients with chronic hepatitis B; the optimum cutoff value of SI was 606, with 74.3% sensitivity and 75.7% specificity, and an area under the curve (AUC) of 0.829 (95% CI 0.737–0.921, *p* < 0.001). (**b**) Receiver operating characteristic (ROC) curves for detecting fibrosis grade in patients with chronic hepatitis B; the optimum ccutoffvalue of SI was 599, with 78.1% sensitivity and 77.5% specificity, and an area under the curve (AUC) of 0.851 (95% CI 0.766–0.935, *p* < 0.001).

A total of 40 patients with CHB were included in this study. The mean patient age was 49.68 ± 10.70 years (19–68). Among them, 20 were female (50%). No patient had a prior history of antiviral therapy for hepatitis. The results of the serum HBV-DNA levels and liver function tests performed on the date closest to the participants’ liver biopsy date are as follows: All patients had HBV-DNA levels above 2000 IU. AST levels ranged from 12 to 96 IU/L (median 38.2 IU/L; reference range: 5–42 IU/L); ALT levels ranged from 10 to 168 IU/L (median 49.45 IU/L; reference range: 5–45 IU/L); albumin levels ranged from 3.44 to 5.10 g/dL (median 4.29 g/dL; reference range: 3.2–5.5 g/dL); total bilirubin levels ranged from 0.34 to 1.01 mg/dL (median 0.65 mg/dL; reference range: 0–1.2 mg/dL); and direct bilirubin levels ranged from 0.05 to 0.2 mg/dL (median 0.11 mg/dL; reference range: 0.001–0.3 mg/dL). Imaging revealed no solid hepatic lesions in any of the patients. The mean duration between hepatic MRI and biopsy was 34 (4–47) days. Before the biopsy, patients exhibited normal coagulation parameters, including INR (<1.5), prothrombin time (PT, <15 s), activated partial thromboplastin time (APTT, <45 s), and platelet counts (>50,000/μL). Needle sizes of 18 G and 16 G were used in 30 and 11 patients, respectively. The total number of pieces was not over three, and a minimum of one piece was obtained from each segment. In one patient, the liver biopsy specimen was deemed insufficient for histopathological evaluation by the pathologist. Maximum and minimum SI values were most commonly obtained in the right lobe in each group. The detection rate of the maximum SI value in the right lobe was higher compared to the minimum SI value (*p* = 0.033). The difference between the mean SIs of the groups was statistically significant (*p* = 0.004). No statistically significant differences were observed between groups for either mHAI or fibrosis scores, including clinically significant mHAI and fibrosis scores (Table 1). Although there was no significant difference between groups, the fibrosis stage was ≥2 in 17 segments (42.5%) and the mHAI level was ≥5 in 20 segments (50%) in group 1; these values were 20 (50%) and 22 (55%), respectively, in group 2. In segments with an SI value < 617, clinically significant mHAI value and fibrosis stage were significantly higher compared to the segments with an SI value > 617 (*p* < 0.001) (Table 2). The ROC results showed that the optimal cut-off value for predicting a SI value for the mHAI was 606, with 74.3% sensitivity and 75.7% specificity (AUC: 0.829, 95% CI 0.737–0.921, *p* < 0.001) and for the fibrosis grade was 599, with 78.1% sensitivity and 77.5% specificity. Moreover, AUC was 0.851 (95% CI 0.766–0.935, *p* < 0.001) (Figure 5a,b). The segmental distribution of liver biopsies is presented in Table 3.

## 4. Discussion

In chronic viral hepatitis, the primary purpose of liver biopsy is to grade and stage liver damage for prognosis and treatment planning. A standard liver biopsy may represent only 1/50,000 to 1/100,000 of the entire liver [15]. Limited tissue sampling, subcapsular sampling, or heterogeneity in the degree of fibrosis between the right and left lobes may lead to misinterpretation in 10–30% of cases [14]. Nearly all studies agree that the diagnostic accuracy of liver biopsy increases with the amount of tissue obtained [15,16]. In our clinical practice, discrepancies between clinical and/or serological findings and histopathological results have been observed in certain cases, leading to diagnostic uncertainty. Based on comprehensive literature reviews on GA-enhanced MRI and our institutional experience, we developed an alternative approach, as we believed that a randomly sampled segment may not reflect overall liver damage. Instead, we performed biopsies from two different segments of the liver that exhibited the highest and lowest signal intensities on GA-enhanced MRI. In each procedure, the number of biopsy samples obtained was limited to a maximum of three, in accordance with our institutional standard protocol. This approach aimed to maximize diagnostic yield while minimizing procedural risk. In our study, we observed that biopsy specimens obtained from segments with low signal intensity (<606) on HBP images in patients with CHB were more likely to show clinically significant inflammation and fibrosis. We believe that pre-biopsy signal intensity measurement on GA-enhanced MRI is a valuable and applicable method for assessing necroinflammation and fibrosis in patients with CHB.

Chronic liver disease and cirrhosis secondary to CHB are common causes of mortality. Morbidity and mortality are directly related to the progression of hepatic fibrosis in these patients [13,18]. It is possible to decelerate the progression and reverse the process of fibrotic remodeling with the treatment of the underlying disease [5,19]. The most important parameter for this is the accurate staging of hepatic pathology. Recently, the non-invasive diagnosis of liver fibrosis has become an important area of study. Some serological indicators and biomarkers have been widely used for this purpose [4]. Aspartate aminotransferase (AST)-to-platelet ratio index (APRI) and fibrosis-4 (FIB-4) are widely accepted reference markers in predicting fibrosis in patients with CHB [4,20]. The combination of AST, anti-HBC, and GGT has shown good prognostic performance in CHB [21]. Fibrosis may affect hepatic function tests; however, these tests cannot detect the regional defects of the liver [12].

US and MR elastography may indicate the stiffness and fibrosis of the liver only indirectly. Both the limited field of vision and operator-dependency limit the optimal hepatic evaluation via US. US can access an area of approximately 1 × 4 cm^3^ in the liver, which corresponds to about 1/500 of the total liver volume [8]. US elastography is the primary method used to assess liver stiffness and provides both qualitative and quantitative information for fibrosis staging. Shear wave elastography (SWE) is the most commonly used quantitative technique for liver elastography. The disadvantages of point SWE include a small ROI, inability to provide real-time imaging, relatively high cost, and higher rates of technical failure and unreliable measurements compared with MRE. Two-dimensional SWE is the most recently developed method of US elastography; although it stands out by providing a larger ROI, real-time imaging, and a color-coded map, a clear superiority of either SWE technique in terms of diagnostic accuracy for staging liver fibrosis has not yet been established [6]. In a single-center prospective study conducted in patients with CHB-related cirrhosis, 2D SWE demonstrated moderate diagnostic accuracy (AUC = 0.72) [22]. In addition, liver stiffness measurements obtained by ultrasound elastography may be adversely affected by necroinflammation and elevated aminotransferase levels [23,24]. Although MRE has several advantages, such as the ability to assess a large portion of the liver, a low rate of technical failure, and the absence of ionizing radiation, it also has some notable disadvantages. The main limitations include technically demanding examination procedures, high equipment and software costs, and technical failures in patients with moderate to severe hepatic iron overload. In addition, conditions such as acute hepatitis with hepatic inflammation, passive congestion secondary to heart failure, and biliary obstruction may lead to overestimation of liver stiffness on MRE [6]. It is impossible to evaluate the whole liver via MR elastography; for example, no measurement can be obtained from the left lobe due to cardiac artifacts [20]. T1 mapping is a novel, multiparametric, contrast-free MRI technique used to assess the degree of liver fibrosis and inflammation. In this method, excessive iron deposition in the hepatic tissue may confound the results by decreasing the measured T1 relaxation time. It has been reported that this technique is equally sensitive to both fibrosis and inflammation, cannot reliably distinguish between different stages of fibrosis, and that larger studies are needed to establish validated cutoff values specific to the stages of fibrosis and inflammation [7]. More recent MRI techniques that show promise for staging liver fibrosis include T1ρ MRI values and DWI-based virtual MRE. However, the association of T1ρ values with the severity of liver fibrosis has only been reported in a very small patient cohort and solely for the right lobe, with no assessment performed for the left lobe. DWI-based virtual MRE, on the other hand, has been shown to improve diagnostic performance in CHB only when combined with serum indices, rather than as a standalone method [1,8]. Each of these noninvasive techniques has distinct advantages and disadvantages in the assessment of liver fibrosis. The accuracy of a single test for evaluating liver fibrosis, particularly in patients with CHB, is limited by multiple factors [7]. In contrast, GA-enhanced liver MRI can be obtained at any center equipped with an MRI scanner. It does not require long or complex procedures, nor high costs. For the assessment of fibrosis, simple placement of ROIs while avoiding blood vessels, bile ducts, and focal lesions is sufficient, without the need for specialized operator experience. Unlike some studies, GA-enhanced MRI has no lobe-specific limitations; measurements can be easily obtained from both the right and left lobes, as well as from all segments. Moreover, since GA exhibits the highest hepatic uptake among contrast agents, it provides additional utility in the evaluation of liver malignancies, including HCC, in which HBV plays a significant etiological role.

GA has been accepted as the primary non-invasive biomarker of hepatobiliary disorders since it potentially indicates hepatocyte function [1,4,11]. Transport of GA to hepatocytes is provided by two different systems located on the sinusoidal and canalicular membranes of the cell [4]. It enters the cell bound to organic anion transporting polypeptide 1 and 3 (OATP1B1 and OATP1B3) and is excreted via multidrug resistance protein 2 (MRP2 = apical transporter) into the bile [12,19,25]. In MRI, peak hepatic SI with GA, which is called HBP, is obtained at the 20th minute after the injection [3]. Imaging patterns of HBP are classified into two categories: hypointense and hyper/iso-intense. These patterns may be explained by the expressions of OATP and MRP. In chronic hepatitis, the number and function of hepatocytes are reduced, and fibrotic tissue accumulation, which prevents access to hepatocytes, is observed. Consequently, OATPB1/B3 activity and contrast agent enhancement are reduced [4]. Starting with this theory, GA-enhanced HBP MRI has been used in many studies to evaluate hepatic function and fibrosis. In the majority of the studies, this evaluation was performed using either direct measurement of hepatic contrast or some quantitative measurements such as relative liver enhancement (RLE), CEI, or T1 relaxation time. Significant outcomes have been reported in almost all of these studies correlating the extent of fibrosis and MRI parameters [5,13,19,26,27,28,29]. Severe fibrosis in CHB has been assessed using the quantitative coefficient of variation parameter in GA-enhanced MRI [4]. There are many studies demonstrating the correlation between the Child-Pugh stage and the degree of parenchymal enhancement [11,30]. However, the most frequently used method for the evaluation of hepatic function in GA-enhanced MRI is the measurement of parenchymal SI, which we have used in our study [3,18,31,32,33]. Additionally, in GA-enhanced MRI, simple SI-based measurements have been proven to be as effective as more complex parameters in evaluating liver function [33].

In chronic hepatitis, there is a strong relationship between the stage and the severity of the disease. Therefore, classification is clinically beneficial for these patients and provides a guide for the management of the disease. The most common pathological grading method is mHAI, also known as Knodell’s score. A score between 1 and 3 was accepted as minimal chronic hepatitis, 4–8 was accepted as mild chronic hepatitis, 9–12 was accepted as moderate chronic active hepatitis, and 13–18 was accepted as severe chronic hepatitis. For the staging, five stages were defined according to the degree of fibrosis and development of cirrhosis (stage 0 = no fibrosis, stage 1 = mild, stage 2 = moderate, stage 3 = severe, stage 4 = active cirrhosis) [17]. Recent guidelines recommend antiviral therapy in moderate and severe inflammations as well [21,34]. In this study, thresholds defined by the Health Practice Guideline prepared according to the EASL criteria were used for the treatment, and ≥5 and ≥2 were accepted as significant for mHAI and fibrosis, respectively.

A strong correlation has been demonstrated between the degree of fibrosis and RLE [3,12,13,19,35]. Moreover, there are studies suggesting overlapping RLE values between different stages of fibrosis. Because hepatic fibrosis progression differs in each segment of the liver, pathological sampling may not reflect the actual fibrosis, and the progression of fibrosis cannot be clearly segmented in the liver. In addition, it has been demonstrated that the carriers in the liver may differ in the absorption or excretion of gadolinium chelate according to their functional capacities [4]. Therefore, uptake and accumulation of the contrast agent would not be the same in different segments of the liver, and a non-homogeneous contrast would be observed in CHB [3,4]. In our study, segmental SI measurements were different, and the maximum and minimum SI measurements were mostly obtained from the right lobe.

Percutaneous liver biopsy is a frequently used and preferred method in the diagnosis and follow-up of liver parenchymal diseases within IR and gastroenterology practice [3,12,18]. Generally, the 6–9th intercostal gap in the mid-clavicular line is preferred for biopsy due to its low risk of complications [36]. However, heterogeneous parenchymal involvement is present in patients with chronic parenchymal disease. The hepatic enhancement measurements have been calculated using the mean ROI measurements obtained from different segments of the liver to reduce the effect of these heterogeneous parenchymal changes. Nevertheless, histopathological sampling is generally performed from a random segment of the liver, which may lead to significant differences in tissue sample results and cause the disease to be diagnosed at a lower stage than it actually is [12,15]. Additionally, there are also risks of sampling errors related to the sample size [4,18]. In CHB, an effective pathological examination requires 11–15 complete portal tracts, which corresponds to a biopsy specimen of at least 20–25 mm in length. Therefore, it is recommended to obtain more than one tissue core during biopsy. On the other hand, it has been shown that performing multiple biopsy passes does not increase the risk of major complications (16). In our daily routine practice, we have been consistently obtaining at least two tissue samples during liver biopsies for quite some time. Additionally, in this study, in which two different segments were biopsied in each patient, we did not encounter any complications. Consequently, there is a need for applying standardized criteria for liver biopsies as a reference procedure [12].

This paper supports the suggestion that GA-enhanced MRI could be included in routine practice as a screening test in the staging of fibrosis in CHB. This approach is easy to use in clinical practice and does not necessitate additional MRI sequences, mathematical modeling, or complex analysis of MRI signal properties. In our study, clinically significant inflammation and fibrosis were observed in biopsy specimens taken from liver segments exhibiting the lowest signal intensity on GA-enhanced HBP MRI (SI cutoff values: 599 for fibrosis grading, 606 for mHAI). Inflammation and fibrosis scores were also significantly higher in liver segments below the median SI value (<617), which supported our cutoff values. Although not statistically significant, only one patient in each group exhibited a fibrosis score of 0/6. For CHB patients with a liver SI value below 606, biopsy of the segment with the lowest SI is recommended, while those with higher SI values may be monitored.

This study includes patients with CHB only because etiology is an effective parameter for parenchymal changes and SI measurements. The patterns of fibrosis may differ according to the underlying etiology of chronic liver disease (1). However, this study had some limitations: It was a retrospective, single-center, and relatively small study. The duration between biopsy and MRI was not standard for each patient. The authors compared the cellular uptake of GA with biopsy only and did not use hepatic function tests, such as the indocyanine green test and non-invasive indexes (APRI, FIB-4), or morphological tests such as elastography. Sample variation may be present since the biopsy of certain segments is difficult. Another relative limitation of the study is the impact of the magnetic field strengths of different scanner brands on the parameters studied. Since this study was conducted at a single center using only one MRI scanner, there was no variation due to differences between MRI devices or centers, and the reproducibility of the measurements was not evaluated. Background steatosis/ iron depositions are known to affect the absolute SI measurements on HBP. However, a recent study concluded that relative enhancement was more consistent over successive examination scanners, and the field strengths and GA-enhanced MRI had various advantages over global liver function tests [37].

## 5. Conclusions

Just as the determination of the biopsy site is critical in large tumors due to the heterogeneity of necrotic tissue, obtaining biopsies from properly targeted sites in the large liver affected heterogeneously by CHB is equally essential for enhancing diagnostic accuracy. Liver biopsies from segments with low SI on GA-enhanced HBP MRI detect clinically significant inflammation and fibrosis more effectively in CHB patients. Therefore, guiding biopsies using SI measurements may improve diagnostic accuracy and enable a more precise assessment of necroinflammation and fibrosis. However, further prospective studies involving larger patient populations with liver parenchymal disease of various etiologies are warranted.

## Figures and Tables

**Table 1:** Radiological findings and comparative pathological outcomes (mHAI and Fibrosis) of biopsies from high-SI (Group 1) and low-SI (Group 2) regions.

	Group 1 (*n* = 40)	Group 2 (*n* = 40)	*p*-Value
Liver segment (right)	31/40	22/40	**0.033**
SI, mean (range)	710.1 ± 231.9 (323–1592)	573.2 ± 175.4 (161–1095)	**0.004**
mHAI grade (≥5)	20/40	22/40	0.654
Fibrosis grade (≥2)	17/40	20/40	0.501
mHAI grade, mean (range)	5.25 ± 2.63 (2–13)	5.28 ± 2.63 (2–13)	0.966
Fibrosis grade, mean (range)	1.70 ± 1.04 (0–5)	1.75 ± 1.06 (0–5)	0.832

A *p*-value < 0.05 was considered statistically significant. *n*: liver segments; *mHAI*: Modified histologic activity index; *SI*: signal intensity.

**Table 2:** Comparison of the two groups formed according to the median SI value of both groups (40 patients, 80 segments).

	SI < 617 (*n* = 40)	SI > 617 (*n* = 40)	*p*-Value
Liver segment (right)	24/40	29/40	0.237
SI, mean	489.5 ± 100.6	793.8 ± 191.1	**<0.001**
mHAI grade (≥5)	30/40	12/40	**<0.001**
Fibrosis grade (≥2)	28/40	9/40	**<0.001**
mHAI grade, mean	6.28 ± 2.47	4.25 ± 2.37	**<0.001**
Fibrosis grade, mean	2.20 ± 1.09	1.25 ± 0.74	**<0.001**

A *p*-value < 0.05 was considered statistically significant. *n*: liver segments; *mHAI*: Modified histologic activity index; *SI*: signal intensity.

**Table 3:** Segmental distribution of liver biopsies.

Liver Segment	Left	Right
2nd	15	
3rd	4	
4th	9	
5th		8
6th		10
7th		17
8th		17
Total	28	52

## Data Availability

The raw data supporting the conclusions of this article is not publicly available but is available from the corresponding author upon reasonable request.

## Abbreviations

CHB	Chronic hepatitis B
HCC	Hepatocellular carcinoma
US	Ultrasound
MRE	Magnetic resonance elastography
ADC	Apparent diffusion coefficient
DWI	Diffusion-weighted imaging
GA	Gadoxetic acid
MRI	Magnetic resonance imaging
CEI	Contrasted enhancement index
ROI	Region of interest
SI	Signal intensity
HBP	Hepatobiliary phase
IR	Interventional radiology
mHAI	Modified histologic activity index
ROC	Receiver operating characteristic
AUC	Area under the curve
PT	Prothrombin time
APTT	Activated partial thromboplastin time
APRI	Aspartate aminotransferase (AST)-to-platelet ratio index
FIB-4	Fibrosis-4
SWE	Shear wave elastography
OATP1B1 and OATP1B3	Organic anion transporting polypeptide 1 and 3
MRP2	Multidrug resistance protein 2
RLE	Relative liver enhancement
